# Enhancing the care of women with rheumatic diseases during pregnancy: challenges and unmet needs in the Middle East

**DOI:** 10.1007/s10067-015-3052-5

**Published:** 2015-08-25

**Authors:** S. Al-Emadi, F. Abutiban, B. El Zorkany, N. Ziade, A. Al-Herz, M. Al-Maini, B. Khan, A. Ghanem, H. Al Rayes, J. Al Saleh, H. Al-Osaimi, M. Østensen

**Affiliations:** Medicine Department Rheumatology Section, Hamad Medical Corporation, PO Box 3050, Doha, Qatar; Department of Rheumatology, Al Jahra Hospital, Al Jahra, Kuwait; Faculty of Medicine, Cairo University, Cairo, Egypt; Department of Rheumatology, Hôtel-Dieu de France and School of Medicine, Saint-Joseph University, Beirut, Lebanon; Department of Rheumatology, Al-Amiri Hospital, Kuwait City, Kuwait; Department of Rheumatology and Immunology, Mafraq Hospital, Abu Dhabi, United Arab Emirates; Mediclinic City Hospital, Dubai Healthcare City, Dubai, United Arab Emirates; Department of Rheumatology, Mubarak Al-Kabeer Hospital, Jabriya, Kuwait; Department of Rheumatology, Prince Sultan Military Medical City, Riyadh, Saudi Arabia; Rheumatology Department, Dubai Hospital, Dubai, United Arab Emirates; King Fahd Armed Forces Hospital, Jeddah, Saudi Arabia; Norwegian National Advisory Unit on Pregnancy and Rheumatic Diseases, St. Olavs Hospital, Trondheim University Hospital, Trondheim, Norway

**Keywords:** Autoimmune rheumatic diseases, Counselling, Interdisciplinary management, Pregnancy, Systemic lupus, Women’s health

## Abstract

Pregnancy in women with rheumatic disorders is known to be associated with risks for both the mother and fetus; however, these risks can be minimized with proper planning and careful management of the disease. In the Middle East, there are specific cultural challenges that may have a negative impact on the care that women with rheumatic disorders receive. There is a need for cross-collaboration between specialist physicians, improved awareness of rheumatic disorders among the general public and more open discussion with patients about the potential complications of pregnancy. Women in the region are often unwilling to discuss their disease with their partner and are even less likely to seek advice regarding family planning from their physician. The objective of this review is to highlight the specific challenges of pregnancy management and to discuss why establishing specialist pregnancy clinics for women with rheumatic disorders could be an effective solution. Such clinics can provide high quality care before, during and after pregnancy as shown in several European and US centers. Additionally, such clinics could be useful for the collection of pregnancy outcomes data from the Middle East, which may currently be lacking in the region, in order to highlight where further improvements can be made. With specialist care and analysis of pregnancy outcomes, the standard of care for women with rheumatic disorders in this area could be significantly improved.

## Introduction

Autoimmune disorders, in particular systemic inflammatory rheumatic diseases (including rheumatoid arthritis [RA], ankylosing spondyloarthritis [AS], systemic lupus erythematosus [SLE], antiphospholipid syndrome [APS] and systemic sclerosis), affect many women, often during childbearing age [1]. Numerous studies have shown that pregnancies in patients with rheumatic diseases are high risk, because of the potential for complications during periods of active disease [2]. Several studies have shown that pregnancy in these patients is associated with poor fetal outcomes [3–5]; for example, higher perinatal mortality rate was observed in mothers with chronic inflammatory arthritides, related to first birth [6]. In previous decades, female patients were sometimes advised against pregnancy due to the risk of disease flares and possible harmful effects of medications on the growing fetus and the newborn child [5]. However, the current opinion is that successful and safe pregnancies are possible if pre-pregnancy planning and screening for maternal and fetal risks are undertaken, and pregnancy takes place while the disease is well controlled [2, 5]. Recent studies have shown improved pregnancy outcomes when pregnancies were planned and managed at specialized high-risk pregnancy clinics [7, 8].

The negative impact of rheumatic diseases on pregnancy outcomes in the Middle East may be greater than elsewhere [9]. In Saudi Arabia, a retrospective evaluation of all pregnancies that occurred in patients with SLE from 1980 to 2006 showed that the proportion of live births was significantly lower (70.2 vs. 85.7 %), and more likely to end in fetal deaths (29.7 vs. 14.2 %) and preterm births (26.7 vs. 5.8 %), in pregnancies occurring after SLE onset than those occurring before [10]. In Egypt, a prospective study reported data on 37 pregnancies in 34 patients with SLE from 2007 to 2009; there were 5 spontaneous miscarriages and 32 pregnancies resulted in live births. However, these births had considerable complications (12 preterm babies; 8 with intrauterine growth restriction; 7 with low birth weight; 6 preterm infants admitted to neonatal intensive care unit, and 4 neonates died) [11]. Pregnancy complications were also reported in a retrospective analysis from 2011 [3]. These observations suggest that there is a need to improve the care and support of pregnant women with rheumatic diseases in the Middle East in order to improve pregnancy outcomes in these patients.

Introducing specialized pregnancy care for women with rheumatic disease is challenging in any country, but physicians in the Middle East face specific challenges that are related to the culture and healthcare systems that prevail in this region. In Qatar, a pregnancy and rheumatic disease clinic was established in 2005. This receives early referrals from rheumatologists and obstetricians, potentially leading to improved outcomes. Establishing specialized pregnancy clinics that are adjusted to the clinical practice in each country could help to provide improved care for pregnant patients.

Based on the experiences of practicing rheumatologists in the Middle East (Saudi Arabia, Egypt, Kuwait, Qatar, United Arab Emirates [UAE] and Lebanon), the objective of this review is to identify the challenges that physicians in the Middle East face when managing patients with rheumatic disorders who are pregnant or of childbearing age, and to discuss how addressing these challenges could help to improve outcomes for patients and their babies.

## Challenges in the Middle East

In the majority of Middle Eastern countries, there is a lack of awareness of rheumatic diseases, as well as their pregnancy-associated complications in the general population. This leads to delayed diagnosis and delayed initiation of treatment [12]. For example, it is not uncommon for a patient to see a rheumatologist for the first time during pregnancy despite having prior disease managed by physicians of different specialties (such as internists, orthopedics and family physicians). Furthermore, lack of expertise regarding rheumatic diseases in other specialties can lead to misleading information and inappropriate therapeutic interventions.

Parallel healthcare systems may also introduce treatment disparities between patients. For example, in the UAE, there is coexistence of private and public healthcare systems: free public healthcare facilities are available predominantly to the local Emirati population while the expatriate population prefers to use private healthcare. In the private healthcare sector, there can be some reluctance from rheumatologists to refer women to pregnancy clinics. In the majority of Middle East countries, both the private and public healthcare systems are coexistent, which can lead to a financial barrier to referral, preventing pregnant patients from receiving optimal care.

An issue that is specific to the private healthcare sector is that there are also inconsistencies in the private health policies provided by different insurance companies and plans. The coverage for chronic conditions, autoimmune diseases, pre-existing diseases, immunotherapy and maternity care vary among different policies. In addition, there are complexities in guidelines followed by various insurance payers; these are often based on clinical guidelines (such as The European League Against Rheumatism [EULAR] or National Institute for Health and Care Excellence [NICE] guidelines), but are influenced by individual policy terms and conditions. Incidences have occurred where pregnant women have been unable to receive the required medical care due to exclusion criteria in their health policies. Furthermore, some patients cannot afford medical costs and are therefore deprived of medical care. These factors pose major challenges in providing optimal care.

Furthermore, it is uncommon for women in the Middle East to seek medical advice while preparing to conceive [13]. For example, participants in a qualitative study of Qatari women’s health beliefs stated that it is not socially acceptable for unmarried women to seek any advice related to pregnancy [14]. Additionally, there is a lack of general health education in some districts. A review of the demographic characteristics of 150 RA patients from five countries in the Middle East found that 34 % of patients (male and female) had either received no formal education or only primary school education [15]. This lack of education can result in misunderstandings; for example, some women believe that their disease will improve when they get married or become pregnant, and as such they do not believe that they need to discuss the possible impact of their disease with their husbands.

Another custom is reluctance to inform the future husband about pre-existing chronic conditions due to fear of marriage cancellation, which could result in delayed access to care in the case of an acute flare during pregnancy. Furthermore, the husband and/or the families can have an influence on the decision of the couple to become pregnant and the timing of the pregnancy. This may eventually interfere with the desire of the couple to seek medical advice when deciding to conceive.

There are a limited number of established patient organisations or support groups within the Middle East, meaning that many women are unaware of the symptoms and the course of rheumatic diseases, and it has been proposed that an educational program to increase public awareness of rheumatic diseases and the spectrum of rheumatology could encourage patients to seek advice with their primary care provider [9] and specialists before planning a pregnancy.

## Data gaps and unmet needs

Currently, there are no collaborations for the collation of data on pregnancy outcomes in the Middle East. Furthermore, there is a lack of epidemiological data regarding rheumatic diseases in general except from the work of the Global Burden of Diseases Working Group [16], and the absence of country-based data can also be a barrier to implementing treatment guidelines for the management of RA [9]. It is important to track pregnancy outcomes of patients with rheumatic diseases to enable comparison with those of the general population so that areas for improvement can be identified. Gathering local epidemiological and health economic data could help to change the perception that RA, and other inflammatory diseases, are low-priority diseases compared with other conditions believed to be more prevalent in the region [9]. It could also raise awareness of the complications involved for pregnancies in women living with these diseases so that coordinated care could be improved. A collaborative approach is likely to be the most successful, as pooling resources would help to ensure that all countries in the area could participate [9].

Due to prevailing healthcare systems, particularly in private sectors, it is very challenging to maintain registries. There is freedom in the private sector to self-refer to specialists and switch doctors, which can lead to difficulties in collecting accurate epidemiological data and impair communication among specialists. In addition, maintenance of registries and follow-up are difficult due to the large turnover of expatriate population moving in and out of various countries of the Gulf Cooperation Council. The lack of patient registries in the region means that doctors cannot make comparisons between data from their clinical practice and the literature.

A systematic review and meta-analysis of pregnancy outcomes in SLE patients gave support for pre-pregnancy counselling and multispecialty care of these patients [17]. A greater availability of epidemiological and statistical data would reinforce the need to establish pregnancy clinics for patients with rheumatic diseases in the Middle East and would encourage greater support from hospital management.

In the first instance, a worthwhile research goal would be tracking pregnancy outcomes in women with rheumatic diseases in the Middle East, which could involve human leukocyte antigen haplotype profiling studies (especially in the presence of arranged marriage in the Middle East or the tradition of marriage among relatives, including cousins) or identifying the regional differences, if any, in pregnancy outcomes in patients with rheumatic diseases. Examples of centers of excellence, which have collected pregnancy outcomes data in women with inflammatory diseases in other countries, include St Thomas’ Lupus Pregnancy Clinic, London, UK [18]; the National Center of Pregnancy and Rheumatic Disease in Trondheim, Norway; the LifeQuest Centre for Reproductive Medicine, Toronto, Canada; the Lupus and APS Center of Excellence, New York, USA; the Obstetric Rheumatology Clinic, Birmingham, UK; and the John Hopkins Lupus Center, Baltimore, USA [19]. Much of the data collected and published from these clinics support the main themes of this report, including the need to counsel women about their options before they become pregnant, and management of these patients by a multidisciplinary team.

Key areas in which other studies have collected data include the following: epidemiological data (including comorbidities or complications, and historical outcomes of the mother and infant), medications, maternal outcomes, fetal/infant outcomes (low birth weight, major congenital malformations, perinatal mortality) and disease outcomes [6, 11, 4]. These data could be collected from Middle East clinics via a standardized data report form, which would capture the range of predefined outcomes outlined above, and could then feed into a central research database for further analysis.

## Enhancing cross-collaboration between specialists

There is a lack of clear national guidelines or expert consensus within many countries in the Middle East relating to the management of patients with rheumatic diseases who are pregnant or are considering pregnancy (Table 1). However, the guidelines that exist in other regions stress the importance of collaborative and consistent care for the best possible outcomes. For example, the UK NICE guidelines for RA state that patients should have access to a multidisciplinary team that provide opportunities for discussion of the effect of their disease on their life [20], such as pregnancy or pre-pregnancy planning. Issues that can be caused by a lack of cross-collaboration include obstetricians designing a pregnancy plan without consulting the rheumatologist, and therefore not considering the various medications that the patient is taking, or obstetricians recommending cesarean sections for pregnant patients when such procedures are not indicated. Data from Norway showed that there was no increase in the rate of emergency cesarean sections in women with inflammatory arthritides above that of the general population, demonstrating that elective cesarean sections are often chosen in anticipation of problems during delivery and might not be necessary [6]. Cross-collaboration between specialists is not common in these fields in the Middle East, which could be due in part to financial motives and/or a lack of expertise. In Qatar, cross-collaboration is well established, and most pregnancies are dealt with by a multidisciplinary team. The presence of a specialized pregnancy clinic raises the awareness of other specialists and may lead to an increase of referrals of pregnant women with rheumatic disease.

**Table 1:** Summary of services and provisions for the care of women with rheumatic diseases in selected countries in the Middle East

Country	Pregnancy clinic?	National recommendations?	Epidemiology data?	Multidisciplinary meetings?	Population^a^	Number of gynaecologists^b^	Number of rheumatologists^b^
Egypt	Yes (1 clinic)	No	No (sporadic reports)	Occasional	80,722,000	Not available	∼1200
Lebanon	No	No	No	Occasional	4,647,000	>500 members LSOG	Lebanese Society of Rheumatology = 53 active members
Kuwait	Planning	No	Kuwait data registry for RA patients (KRRD)	No	3,250,000	Not available	Kuwait Association of Rheumatology = 27^c^
UAE	Yes	No	Yes (Abu Dhabi)	No	9,206,000	Not available	70
Saudi Arabia	Planning	No	No	No	28,288,000	Not available	120
Qatar	Yes	No	Data registry for RA patients	Yes	2,051,000	Not available	12

^a^Population in 2012. World Health Organization website. Available at: www.who.int (accessed 18/7/14)

^b^Number of gynecologists and rheumatologists estimated based on personal communication with rheumatologists

^c^Does not include private practitioners

In order to achieve better patient care and improve outcomes, a centralized process for coordinated management of pregnant patients with rheumatic diseases would encourage cross-collaboration. This would potentially involve establishing dedicated regional centres where a standard of care could be ensured for all patients. The aim should be to ensure that patients receive consistent advice from all specialties involved in their care and that women are treated with both their pregnancy, or future pregnancy, and their disease in mind. It should be emphasized that men with rheumatic diseases also need counselling while considering starting a family as certain drugs can have an impact on fertility.

## Establishing pregnancy clinics for patients with rheumatic disorders

Ideally, women with rheumatic diseases who become pregnant, or who wish to become pregnant, should be seen in specialized clinics for high-risk pregnancies. A small number of specialist clinics have been established in hospitals in Qatar and Saudi Arabia, but the majority of countries in the Middle East do not yet have these clinics available. The authors of this report, the majority of whom are rheumatologists practicing in the Middle East, believe that such clinics would be valuable additions to their healthcare systems; helping to increase awareness of rheumatic diseases, and improve patient counselling and care during pregnancy.

**Fig. 1:**
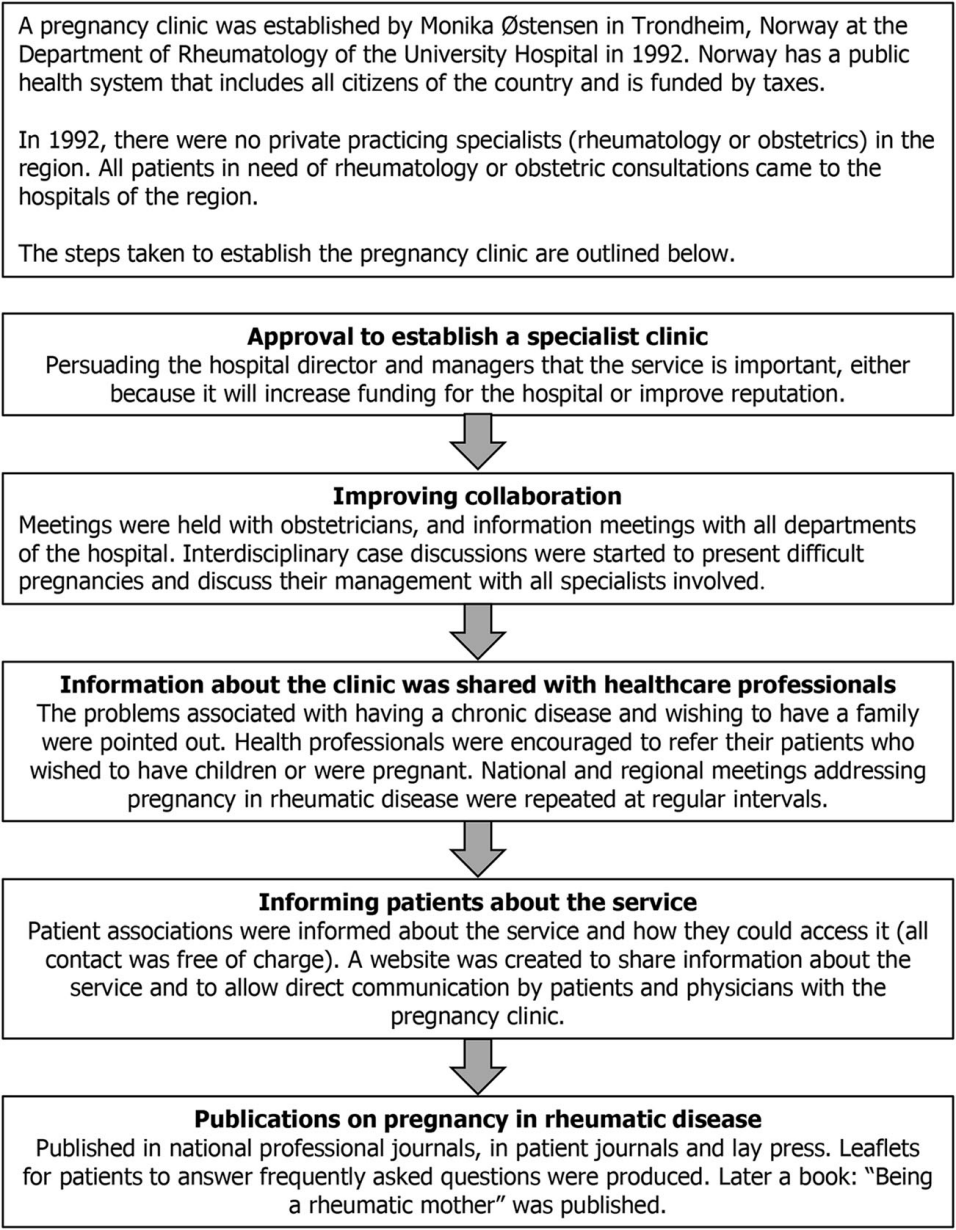
Establishing a pregnancy clinic: a case study example

The key challenges to establishing these clinics are cross-collaboration, education of medical staff and patients, and support from management. Based on one author’s experience, problems encountered when establishing a pregnancy clinic in Trondheim, Norway, included difficulties convincing the hospital management that pregnant patients with rheumatic disease deserve a specialized service when this may not initially be cost-effective. While it was easy to persuade primary care physicians that such a clinic would be helpful, as in general they did not feel competent to deal with patients with high-risk pregnancies, convincing other specialists proved to be more difficult. The key steps that were taken to establish a successful clinic are outlined in Fig. 1. Furthermore, setting up a clinic in Berne, Switzerland, presented different problems, due to the different healthcare system in place (public and an extensive private healthcare system versus a tax-funded public healthcare system in Norway). The specialists in private practice were reluctant to refer their pregnant patients out of fear of losing income. In order to address this, the team had to guarantee that the patient would remain in the specialists’ care and only have consultations at the clinic for the duration of the pregnancy.

## Consultation best practice

**Fig. 2:**
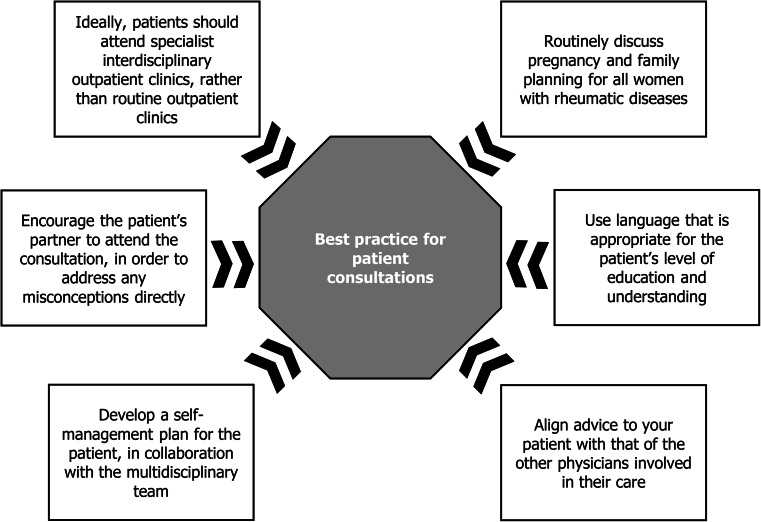
Considerations for best practice in consultations for patients with rheumatic diseases

If it is not possible to establish a pregnancy clinic, rheumatologists and obstetricians can also help to improve the care of women with rheumatic diseases by following best practice during consultations with these women (Fig. 2). Pregnancy and family planning should be discussed routinely with all women with rheumatic diseases that are of childbearing age, using language that is suitable for the patient’s level of understanding. It should not be assumed that older patients, those who already have children, or women who have re-married do not want to have further children.

Women with rheumatic diseases should undergo counselling before conception for their specific risk profile [5], and subsequently a management plan should be developed. It is also important for physicians to respect that it is the patient’s right to choose to become pregnant, and to support them in this decision regardless of the physician’s own personal beliefs.

Where possible, women with rheumatic diseases who would like to discuss pregnancy should attend medical appointments with their partners. There is evidence that the moral support from family members contributes to an increased use of maternal health services by women in rural Egypt [21], and, in some regions, women often have strong beliefs that they must follow the guidance of their husband rather than their physician. If partners attend appointments together, misapprehensions can be discussed and addressed.

It may be helpful for the treating physician to review other sources of information the patient has accessed in order to mitigate any harmful misunderstandings or advice. In the UK, the NICE Quality Standard for RA [22] recommends that education and self-management should be employed to improve a patient’s understanding of their disease. Additionally, it states that this education should be offered throughout the disease course and should be individualized to the patient; therefore, education offered to women of childbearing age should include advice about their disease and the possible implications and management strategies during pregnancy. Although these recommendations are specific to RA, such considerations could also be applied more widely to other systemic inflammatory conditions that may impact pregnancy outcomes.

## Conclusions

As compared to other parts of the world, cultural and traditional factors may affect the health system and care of pregnant women with rheumatic disease in the Middle East. These factors should be carefully studied and approached to improve the standard of care offered to women in this area.

Communication between specialists is essential to ensure that patients receive the highest standard of care prior to, throughout and after pregnancy. Cross-collaboration between specialists should be encouraged and supported by management where possible, and establishing specialized pregnancy clinics in the Middle East region involving these multidisciplinary teams could help to ensure the best possible outcomes for both mother and infant. However, where this is not possible, the primary goals of physicians should be to ensure that women with rheumatic diseases receive pre-pregnancy counselling, are referred appropriately and are supported throughout pregnancy. The core emphasis should be to increase awareness among the general population of the need for this service and provide access to the nearest available specialized pregnancy clinic for women and men with rheumatic diseases.
